# *Chenopodium Ambrosioides* Linn Mitigates Bone Loss in Rats with Periodontitis

**DOI:** 10.30476/dentjods.2023.95767.1891

**Published:** 2024-03-01

**Authors:** Diego Thiers Oliveira Carneiro, Michael Douglas da Silva, Karla Vanessa Pinto Vasconcelos, Romero Dias, Vanessa Costa, Raquel Felipe Vasconcelos, Bárbara Carneiro, Gisele Barreto, Mirna Marques, Hellíada Chaves Vasconcelos, Howard Lopes Ribeiro Júnior, Jonas Nogueira Ferreira Maciel Gusmão, Helson da Silveira, Renata Leitão, Gerly Anne Brito, Karuza Maria Alves Pereira, Delane Viana Gondim, Paula Goes

**Affiliations:** 1 Dept. of Morphology, School of Medicine, Federal University of Ceará, Fortaleza, Brazil; 2 Post Graduation Program of Dentistry, School of Dentistry, Federal University of Ceará, Fortaleza, Brazil; 3 Post Graduation Program in Health Science, School of Medicine, Federal University of Ceará, Sobral, Brazil; 4 Dept. of Pathology and Legal Medicine, School of Medicine, Federal University of Ceará, Fortaleza, Brazil

**Keywords:** Periodontitis, Inflammation, *Chenopodium ambrosioides*, Flavonoids, Rutin

## Abstract

**Statement of the Problem:**

Periodontitis is an inflammatory disease that causes bone loss. Some patients do not respond well to the classic treatment and need therapies that minimize bone loss, the main sequel of the disease. *Chenopodium ambrosioides* L. has stood out due to its anti-inflammatory and anti-oxidative activities. However, no study has yet investigated its effect on periodontitis.

**Purpose:**

This study aimed to evaluate the bone protective effect of *Chenopodium ambrosioides* L. (CAL) extract on ligature-induced periodontitis model in rats.

**Materials and Method:**

For this, a pre-clinical assay was performed, using male Wistar rats divided into 3 groups: Naive (N) (n=6), not submitted to any procedure; Saline (SAL) (n=6), submitted to ligature-induced periodontitis and receiving 2 ml/kg of 0.9% saline solution; and CAL extract, which was subdivided into 3 subgroups (n=6/subgroup) receiving the CAL at 3 (CAL3), 10 (CAL10) or 30 mg/kg (CAL30). All agents were given, by oral gavage, 30 min before periodontitis induction and daily until euthanasia (11^th^ day). By then, maxillae were removed for macroscopic, histological, and histometric analyses. Kidneys, liver, and stomach were collected to evaluate the safety of CAL extract. High-performance liquid chromatography (HPLC) assay was used to investigate the flavonoid content in the extract.

**Results:**

*Chenopodium ambrosioides* L. extract at 30mg/kg showed a reduction by 58% in bone loss marked by an increase (+35%) in the number of osteoblasts and a reduction (-51%) on the number of osteoclasts (*p*< 0.05). No significant alteration in the liver, kidney, or stomach was seen. Rutin was the main flavonoid found.

**Conclusion:**

In summary, it was observed that *Chenopodium ambrosioides* L. extract has shown important anti-inflammatory and bone anabolic and anti-resorptive properties without causing toxicity in the main organs. Rutin, as the main flavonoid of the extract, seems to be responsible for the beneficial effect of this agent.

## Introduction

Periodontitis is one of the most prevalent worldwide chronic inflammatory diseases that compromise the supporting tissues of tooth. Biofilm accumulation is understood as the initial etiologic factor for periodontal destruction. Following, host immune response, marked by the activation of immunological cells and fibroblasts, stimulates the release of several inflammatory molecules that induce tissue destruction and leading to resorption of the alveolar bone [ 1 ].

The mechanical removal and control of the biofilm has been considered the main goal of the periodontal treatment [ 2 ]. Nevertheless, for some individuals, only classical therapies itself are not enough in order to control the disease progression. Thus, it becomes necessary the use of adjunctive therapies [ 3 ]. Pharmacological therapy has been widely used for bone diseases, however the use of natural products has stood out, in comparison to the pharmacological methods due to its high availability, low costs, and lower side effects [ 4 ].

Therefore, the use of plant-derived chemicals has been considered an interesting alternative therapy instead of using to synthetic chemicals. In February 2009, the Ministry of Health from Brazil, presented a List of Medicinal Plants of Interest to the Health Unique System [ 23 ]. Among them, there is *Chenopodium ambrosioides* L. (Mastruz) which one of the most used herb in the general population. *Chenopodium ambrosioides* L. is an herb that belongs to the Amaranthaceae family. It is well distributed throughout Brazil, and in folk medicine, the plant must be crushed and taken in the form of teas, infusions, or poultices for different purposes. Studies have proven its anti-oxidant and anti-inflammatory [ 5 - 6 ] activities are mainly due to flavonoids, such as rutin, myricetin, and quercetin [ 6 ].

In the bone tissue, *Chenopodium ambrosioides* L. extract has prevented bone loss in ovariectomy-induced osteoporosis [ 7 ] and in collagen-induced arthritis through reduction of IL-6 and TNF [ 8 ] and also induced bone repair in a rodent model [ 9 ]. Despite these data, studies focusing on the effect of *Chenopodium ambrosioides* L. extract on periodontitis are still lacking. Thus, considering the anti-inflammatory and anti-oxidant effects of *Chenopodium ambrosioides* L, this study aimed to investigate the efficacy of *Chenopodium ambrosioides* L extract in alveolar bone tissue of rats submitted to a periodontitis model.

## Materials and Method

### Study design and ethical aspects

This was a prospective randomized, controlled, and blind study using animal models. Randomization was performed by computer based on the body weight of the animals. In all analyses, the examiner was blinded to the group. The experimental protocols followed the ARRIVE guidelines for the use of animals [ 10 ]. The assays were initiated only after Institutional Ethics Committee approval from UFC (#149/2017).

### Animal selection

Sixty male Wistar rats (*Rattus novergicus*), 12-weeks-old, weighing 200 g were used. These animals were derived from the Central Animal Facility at the Federal University of Ceará (UFC). They all received free food and water and they were kept in the same environmental condition of a 12/12 h light cycle and room temperature of 22°C during the experiment.

### Ligature-induced periodontitis model

In this study, it was used the model of ligature-induced periodontitis based in Goes *et al*. [ 11 ]. Briefly, the model consists of inserting a 3.0 nylon threat around the second upper left molar of the rat anesthetized with Ketamin and Xylazin (80 mg/kg of Ketamin + 10 mg/kg of Xylazin; ip.). Following, a surgical knot was given facing the vestibular side of the rat’s mouth. The animals were accompanied daily, and after 11 days, they were euthanized by an overdose of anesthetics (three times more than the dose used for anesthesia).

### Experimental Groups

The animals were divided as follows:

● **Naive (N):** a group of 6 animals not submitted to any treatment;

● **Saline (SAL):** a group of six animals submitted to ligature-induced periodontitis that received 2 ml/kg of 0.9% saline solution, by oral gavage, 30 minutes previously to the ligature induction and daily until euthanasia on day 11.

● ***Chenopodium ambrosioides Linn* Groups (CAL):** The animals were subdivided into three groups (n=6/each). All of them were submitted to ligature-induced periodontitis. They received the hydroalcholic extract from the leaves of *Chenopodium ambrosioides* L. diluted in 0.9% saline solution in 3 different doses according to the subgroup (CAL3= 3mg/kg; CAL10= 10mg/kg; CAL30= 30 mg/kg) by oral gavage, 30 minutes previously to the ligature i-nduction and daily until euthanasia on day 11 [ 12 - 14 ].

60 animals were used, divided into 2 sets of experiments. Set 1 was used for macroscopic study and Set 2 for histological analyses. In each set of experiments, 30 animals were used, divided into 5 groups with 6 animals in each group.

### Preparation of the hydroalcoholic extract of *Chenopodium ambrosioides* Linn

The protocol used was based on Silva *et al*. [ 15 ]. Briefly, the plant sample was dried at 38 °C in a stove and then submitted to trituration. The obtained material was submitted to fractional maceration with 70% ethanol, for 24 hours, 4 times. The material was filtered and submitted to pressure. The concentrated dry extract was then diluted in distilled water to be used in the biological assays. 

### Morphometric analysis of bone tissue

By the end of the experiment, the animals were euthanized, and the animals had their maxilla collected and fixed in 10% formalin for 48 h. Later, the maxillae were separated in half, dissected, and 1% methylene blue was used to stain the specimens [ 11 ]. For quantification of bone loss, both hemimaxillae were photographed together with a known area in order to convert pixels into mm^2^. Image J® software (NIH, Bethesda, USA) was used to quantify the alveolar bone loss (ABL) area. The resorption area was calculated considering the space between the top of molars tips until the remaining bone border, subtracted by the respective contralateral area from the animal itself. This area was represented in mm^2^ [ 11 ]. 

### Histopathological analysis of periodontal tissue

In another set of experiments, on the 11^th^ day, the maxillae were collected and fixed in 10% buffered formalin for 48 h and demineralized in 10% EDTA for 30 days. Later, the specimens were embedded in paraffin, and 4µm-thickness sections were collected. The interproximal area between the first and second molar in a mesial-distal direction was used for evaluation. The slides in H&E staining were analyzed under a light microscope at 40X magnification. The inflammatory infiltrate, and integrity of bone and cementum were evaluated following the criteria described by Leitão *et al*. [ 16 ], Score 0: None or slight inflammatory infiltrate (inflammatory cells sparse and restricted in the marginal gingival region), alveolar process and preserved cementum. Score 1: Inflammatory infiltrate moderate (inflammatory cells present throughout the entire length of the gingiva), small bone resorption and cementum without alterations. Score 2: Marked infiltration of inflammatory cells in gingiva and periodontal ligament, great destruction of the alveolar bone and partial destruction of cementum. Score 3: Marked inflammatory infiltrate, intense alveolar bone loss, and severe cementum destruction.

### Histomorphometric analysis of alveolar bone

After histopathological analysis, five fields at 400× magnification were selected in the interproximal region between the first and second molar to count the number of osteoblast/bone perimeter (N.Ob/B.Pm). Another 4µm section from the previously obtained paraffin blocks was collected and stained with HYPERLINK "https://pubmed.ncbi.nlm.nih.gov/18365835/" Tartrate-resistant acid phosphatase (TRAP) for visualization of osteoclast and count the number of osteoclast/bone perimeter (N.Oc/B.Pm). ImageJ® Software (NIH, Bethesda, USA) performed the cell counts [ 17 ].

### Analysis of toxicity in stomach, liver, and kidneys

After euthanasia, the animals' stomach, liver, and kidneys were removed, weighed, and fixed in 10% formalin for 48 h. In sequence, the organs were embedded in paraffin and H&E was used for staining. Morphological analysis was performed using light microscopy, to evaluate a possible toxic effect of the extract. For each organ, specific morphological structures were analyzed using scores: Score 0: Histopathological characteristic absent; Score 1: Mild histopathological characteristic; Score 2: Moderate histopathological characteristic; Score 3: Severe histopathological characteristic [ 18 - 19 ].

In the stomach, the region of the cardia, the presence of ectatic vessels and tissue bleeding were evaluated .Moreover, in the back part of the stomach, the presence of gastric mucosal glands, ectatic vessels, tissue bleeding, inflammatory infiltrate, loss of cells and swelling were assessed. The portal vein and lobular central vein congestions, edema in hepatocytes, sinusoidal bleeding, and inflammatory infiltrate were evaluated in the liver. Edema and vacuolization of the cells from tubular epithelium, recent interstitial and tubular bleeding, nephrotoxic necrosis, hyaline cylinder, and loss of cells were evaluated in the kidney [ 18 - 19 ]. 

### Extract analysis by High Performance Liquid Chromatography (HPLC)

The extract was analyzed by HPLC using an analytic column Phenomenex RP-C_18_ (250 x 4.6mm). The solvents were filtered by a PIFE nylon membrane with pores of 0.45 μm. Samples were dissolved with solvents used on the mobile phase and filtered with PTFE/B 0.45 μm filter. The identification of Rutin and Quercentin was performed by comparing the peak retention time (t_R_) with a reference compound [ 20 ].

### Statistical analysis

Shapiro-Wilk test was used to check the normality of the data. Parametric data were presented as mean± standard error of the mean (SEM), and the ANOVA test was used, followed by the Tukey test. Non-parametric data were presented as median (range) using Kruskal-Wallis followed by the Dunn test. In all situations, the significance level was *p*< 0.05. GraphPad Prism®, version 6.0 was used for all the analysis. 

## Results

### *Chenopodium ambrosioides* extract mitigates bone loss and modulates inflammation

**Figure 1:**
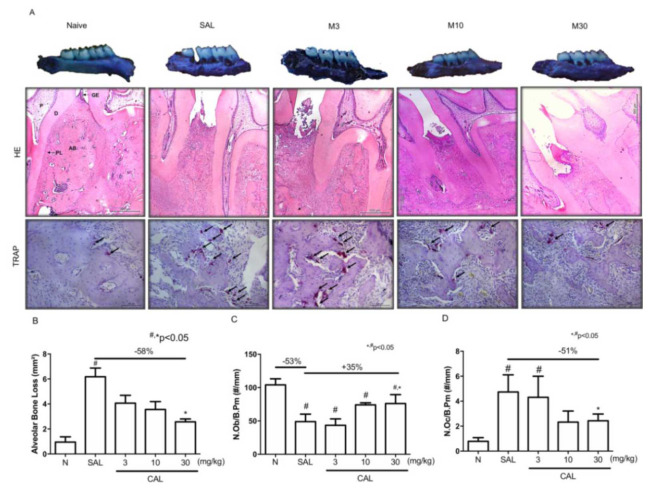
*Chenopodium ambrosioides* extract mitigates alveolar bone loss. **A:** macroscopic and histological aspects (HE and TRAP staining) of hemimaxillae of animals from Naive, SAL, CAL3, CAL 10, CAL30 groups **B:** Alveolar bone loss; **C:** Number of osteoblasts/Bone perimeter (N.Ob/B.Pm); **D:** Number of osteoclasts/Bone perimeter (N.Oc/B.Pm). Data represent the mean±SEM of six animals per group. Statistical analyses were performed by ANOVA followed by the Bonferroni test. (#) *p*< 0.05 when compared to Naive (*) *p*< 0.05 when compared to SAL, magnification of 40×, 400× **G:** Gingiva, **D:** Dentin, **P:** Pulp, **PL:** Periodontal Ligament, **AB:** Alveolar bone

The macroscopic analysis indicated that the periodontitis model induced by ligature caused great alveolar bone loss (Figure 1a-b), marked by furcation lesion and root exposure (Figure 1a) when compared to the maxilla of the Naive group (Figure 1a). The treatment with *Chenopodium ambrosioides* L extract at 3 or 10 mg/kg was not able to prevent bone loss (Figure 1a-b). In the other hand, at 30 mg/kg, *Chenopodium ambrosioides* L. extract significantly reduced bone loss when compared to the SAL group (Figure 1a-b) with preservation of the alveolar bone and less extension of furcation lesion (Figure 1a).

The histopathological analysis confirmed the macroscopic findings. Periodontitis caused intense inflammatory infiltration and loss of bone (Figure 1a; Table 1) compared to the Naive group (Figure 1a; Table 1). In the group that received Saline solution, it was also observed a reduction in the number of osteoblasts (*p*<0.05) (Figure 1c) and an increase in the number of osteoclasts

**Table 1:** Histological analysis of periodontium of animals submitted to periodontitis and treated with *Chenopodium ambrosioides* extract

Experimental Groups					
	Naive	Sal	CAL3	CAL10	CAL30
Scores	0(0-1)	3(3-3)#	3(2-3)#	1(1-2) *	1(1-2)*

Sal: Saline group

CAL= *Chenopodium ambrosioides*

# : Indicates difference compared to N group

* : Indicates difference compared to DP group

Kruskal-Wallis and Dunn test (*p*< 0.05)

(Figure 1a and 1d) compared to the Naive animals (*p*< 0.05). The treatment with CAL at 30 mg/kg significantly increased the number of osteoblasts (Figure 1c) and reduced the number of osteoclasts (Figure 1d) compared to SAL.

### *Chenopodium ambrosioides* extract did not cause toxicity

**Figure 2:**
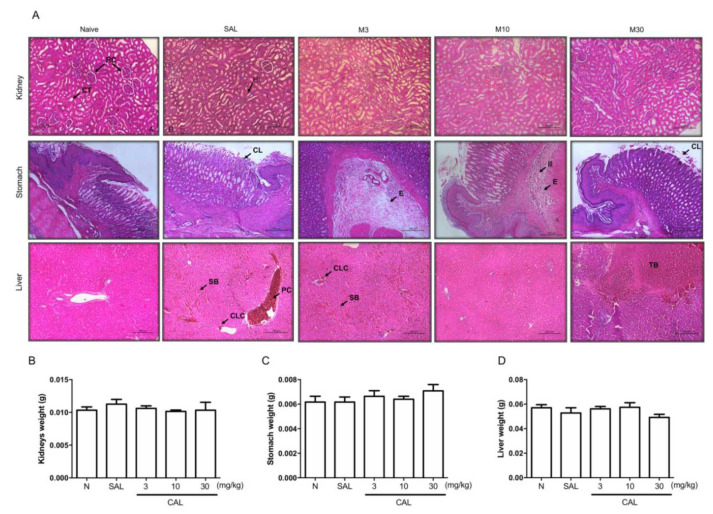
*Chenopodium ambrosioides* extract did not cause important change on kidneys, stomach or liver, A: Representative images in H&E staining of Kidney, Stomach and Liver of animals from Naïve, Saline, CAL3, CAL10 and CAL30 in 10x magnification. B: Kidney weight; C: Stomach weight; D: Liver weight Data represent the mean±SEM of six animals per group. Statistical analyses were performed by ANOVA followed by the Bonferroni test (*p*< 0.05). RC: Renal corpuscle; CT: Contorted tube; CL: Cell loss; E: Edema; II: Inflammatory infiltrate; SB: Sinusoidal bleeding; PC: Portal congestion; CLC: Centre lobular congestion; TB: Tissue bleeding

In the kidneys, mild edema and vacuolization in the tubular epithelium were observed, along with moderate tubular bleeding in animals submitted to periodontitis. The same findings were seen after the treatment with *Chenopodium ambrosioides* extract (Figure 2a; Table 2). Neither periodontitis nor the extract doses affected the weight of the kidneys (Figure 2b). 

**Table 2:** Histological analysis of Liver, Stomach and Kidney of animals submitted to periodontitis and treated with *Chenopodium ambrosioides* extract

Histopathological Aspects	Experimental Groups				
	Naive	SAL	CAL 3	CAL 10	CAL 30
**Liver**					
● Portal and center lobular Vein congestion	0 (0-2)	1 (1-2)#	1 (0-1)	0 (0-2)*	1 (0-2)
● Edema of hepatocytes	0 (0-1)	1 (0-1)#	0 (0-1)	1 (0-1)	1 (1-2)
● Sinusoidal bleeding	1 (0-2)	2 (1-3)#	1 (0-2)	1 (1-1)*	1 (1-2)
● Inflammatory infiltrate	0 (0-0)	1 (0-1)	1 (1-2)	1 (0-1)	0 (0-1)
**Kidneys**					
● Edema of cells from tubular epithelium	0 (0-1)	1 (1-2)	1 (1-2)	1 (1-2)	1 (1-2)
● Vacuolization of cells from tubular epithelium	0 (0-1)	1 (1-2)	1 (1-2)#	1 (1-2)	1 (1-2)
● Recent interstitial and tubular bleeding	1 (0-2)	2 (1-2)	1 (1-2)	1 (1-2)	1 (1-2)
● Nephrotoxic necrosis	0 (0-0)	0 (0-0)	0 (0-0)	0 (0-0)	0 (0-0)
● Loss of cells	0 (0-1)	0 (0-2)	0 (0-2)	1 (0-2)	1 (0-1)
**Stomach-Cardia**					
● Ectatic vessels	0 (0-1)	1 (0-1)	0 (0-1)	1 (1-1)	1 (0-1)
● Bleeding	0 (0-0)	0 (0-0)	0 (0-0)	0 (0-0)	0 (0-0)
● Presence of gastric mucosal glands in	0 (0-0)	0 (0-0)	0 (0-0)	0 (0-0)	0 (0-0)
**Stomach–back**					
● Ectatic vessels	1 (0-1)	1 (0-1)	1 (0-1)	1 (0-1)	1 (1-1)
● Bleeding	0 (0-0)	0 (0-0)	0 (0-0)	0 (0-0)	0 (0-0)
● Inflammatory Infiltrate	0 (0-1)	1 (0-1)	1 (0-2)	1 (0-1)	1 (0-1)
● Loss of cells	0 (0-1)	0 (0-2)	0 (0-1)	0 (0-1)	0 (0-2)
● Edema	0 (0-0)	1 (0-1)	1 (1-2)#	1 (1-2)#	1 (0-1)

Sal: Saline group

CAL: *Chenopodium ambrosioides*

# : Indicates difference compared to N group

* : Indicates difference compared to DP group

Kruskal-Wallis and Dunn test (*p*< 0.05).

The stomach of the animals from the periodontitis group showed mild ectatic vessels in the cardia and back regions and mild inflammatory infiltrate and edema. Using the extract of *Chenopodium ambrosioides* did not change these findings (Figure 2a and Table 2). No change was seen in stomach weight (Figure 2c).

Considering the liver of the animals with periodontitis, moderate portal and lobular center veins congestion was seen and discrete edema in the hepatocytes with moderate sinusoidal bleeding, in the absence of inflammatory focus. The treatment with the extract of *Chenopodium ambrosioides* mitigated these findings, mainly at 10mg/kg (Figure 2a and Table 2). Liver weight was not altered by either periodontitis or the extract (Figure 2d).

### The extract of *Chenopodium ambrosioides* L. presents Rutin peaks

**Figure 3:**
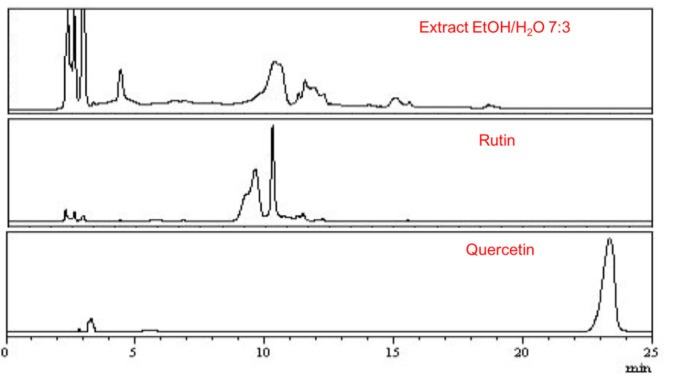
High performance liquid chromatography of *Chenopodium ambrosioides* L. extract and Rutin and Quercetin standards. The retention time of the extract occurred between minutes 9 and 13. Rutin retention time happened between minutes 9-13. Quercetin retention time was visible between minutes 22-24

The analysis of the *Chenopodium ambrosioides* L. extract by HPLC demonstrated that the retention time for flavonoids in the extract occurred between minutes 9 and 13, compatible with the Rutin retention time used as standard (minute 9-13) but different from the retention time of Quercetin, the other standard (retention time minute 22-24) confirming the high level of the flavonoid Rutin in the extract (Figure 3).

## Discussion

This study demonstrated that the model of ligature-induced periodontitis caused intense bone loss and inflammation, reproducing the main findings of human periodontitis. On the other hand, the extract of *Chenopodium ambrosioides* L. reduced bone loss and mitigated inflammation, reducing the inflammatory infiltrate on the periodontium. The extract did not cause toxicity in the liver, kidney, or stomach of the animals. The constituent analysis of the extract revealed high levels of the flavonoid Rutin.

*Chenopodium ambrosioides* L. is known as “mastruz”. This plant is widely found in South America and well distributed in Brazil, and has been used, in folk medicine, to treat rheumatic, inflammatory, helminthic, fungal diseases [ 21 - 22 ]. Therefore, in 2009, *Chenopodium ambrosioides* L was included in the list of National Medicinal Plants of Interest in Brazil [ 23 ], indicating the need to better understand the biological mechanisms responsible by the pharmacological potential of this plant, since studies using C. ambrosioides L. are scarce.

This study showed that the highest dose of *Chenopodium ambrosioides* L. extract significantly reduced bone loss. This finding is in accordance with previous studies that demonstrated that *C. ambrosioides* L. extract prevented bone loss in periodontitis [ 7 - 8 ] and stimulated bone neoformation in bone defects induced in the tibia of Wistar rats with a diameter of 2 mm [ 9 ]. 

The bone protective effect of C. ambrosioides L. extract seen in this study was marked by the increase in the number of osteoblasts with a reduction in the osteoclast number. It has been shown that the extract of C. ambrosioides L. accelerated and stimulated pre-osteoblast proliferation and activity [ 9 ], confirming our results.

The HPLC analysis performed in this study revealed high levels of Rutin in the C. ambrosioides L. extract. Phytochemical analysis of C. ambrosioides L. has indicated the presence of cardiotonics and anthraquinones, alkaloids, tannins, and especially flavonoids [ 20 ]. Among the different compounds, Rutin stands out as the main flavonoid found in the extract [ 6 ].

In bone tissue, Rutin induces osteoblast differentiation by the increase in the expression of both the Runx2 gene and protein [ 24 - 25 ], as well as osteocalcin levels [ 26 ], alkaline phosphatase (ALP) activity [ 24 - 26 ], collagenogenesis and mineralization [ 26 - 27 ] in osteoblast cell-like culture. ALP is an early osteogenic differentiation marker. Runx2 is a specific transcriptor factor for osteoblast differentiation, while osteocalcin reflects osteoblast function and is a late indicator of osteogenic differentiation, which explains the bone anabolic activity of *C. ambrosioides* L. extract. *Chenopodium ambrosioides* L. extract also markedly reduced the number of osteoclasts. It has been reported that Rutin downregulated the expression of osteoclast activity markers (CA2, CALCR, and CTSK) in *in vitro* assay [ 27 ]. Signaling pathways that orchestrate bone metabolism, such as PI3K/AKT/mTOR [ 25 ] and Wnt, have been pointed out as the ones activated by Rutin. However, more studies are needed to clarify its mechanism of action.

This study also revealed that *C. ambrosioides* L. extract modulated inflammation in the periodontium, confirming the widely known anti-inflammatory activity of *C. ambrosioides* L. Previous studies have shown that *C. ambrosioides* L. extract reduced neutrophil and monocytes count on blood [ 8 ], as well in inflammatory infiltrate on bone tissue [ 9 ] acting on the activation and recruitment/proliferation of immune cells, modulating inflammation. *C. ambrosioides* L. extract also reduced serum levels of IFN, IL-6, TNF-a, and IL-17 [ 8 ]. Rutin has well-known anti-inflammatory activity. It has been reported that Rutin significantly reduced IL-1β, IL-6, and TNF-α serum levels, in rats submitted to ovariectomy [ 28 ]. These cytokines are crucial in the pathophysiology of periodontitis, promoting osteoclast differentiation and bone resorption [ 29 ]. 

Despite the excellent local bone properties, it is extremely important to evaluate the systemic safety of the extract. This study analyzed the effect of C. ambrosioides L. extract in the liver, kidney, and stomach. No alteration was seen in the kidneys and stomachs of the animals after the use of the extract. Interestingly, a slight alteration in the liver histological analysis was observed in the animals submitted to periodontitis. Tomofuji *et al*. [ 30 ] reported changes in the liver of animals submitted to ligature-induced periodontitis. PMNs generate excessive amounts of reactive oxygen species in periodontal lesions that enter the blood circulation and oxidize circulating lipids. Despite the low toxicity, circulating lipid peroxide remains within the body for a long time and can gradually affect multiple organs, including the liver [ 31 ].

The current study has some limitations. No data were collected concerning the blood level of pro-inflammatory cytokines or specific osteogenic markers. Moreover, despite showing the same findings of human disease, mainly bone loss, ligature-induced periodontitis does not represent all aspects of human periodontitis, especially considering the critical role of biofilm as a trigger. Therefore, ligature-induced periodontitis is an effective method for exploring its mechanisms [ 32 ]. Still, studies using other periodontitis models, such as LPS-induced periodontitis, are essential to understand the interaction of host/pathogen. Finally, the analysis of Rutin should be performed in the future to confirm its effect on periodontal tissue.

## Conclusion

In conclusion, *C. ambrosioides* L. extract, marked by the high levels of Rutin, showed bone anabolic and anti-resorptive properties, as well as anti-inflammatory activity without toxic effects, being a potential therapeutic tool to be used as an adjunctive to periodontal treatment.
